# Supplementary material for: Characterization of JAK1 Pseudokinase Domain in Cytokine Signaling
Source: Cancers (Basel). 2019 Dec 27;12(1):78. doi: 10.3390/cancers12010078 (PMC7016850; doi:10.3390/cancers12010078)
Supplement: Supplementary file 1 [file cancers-12-00078-s001.pdf]

Supplementary: Figure 1

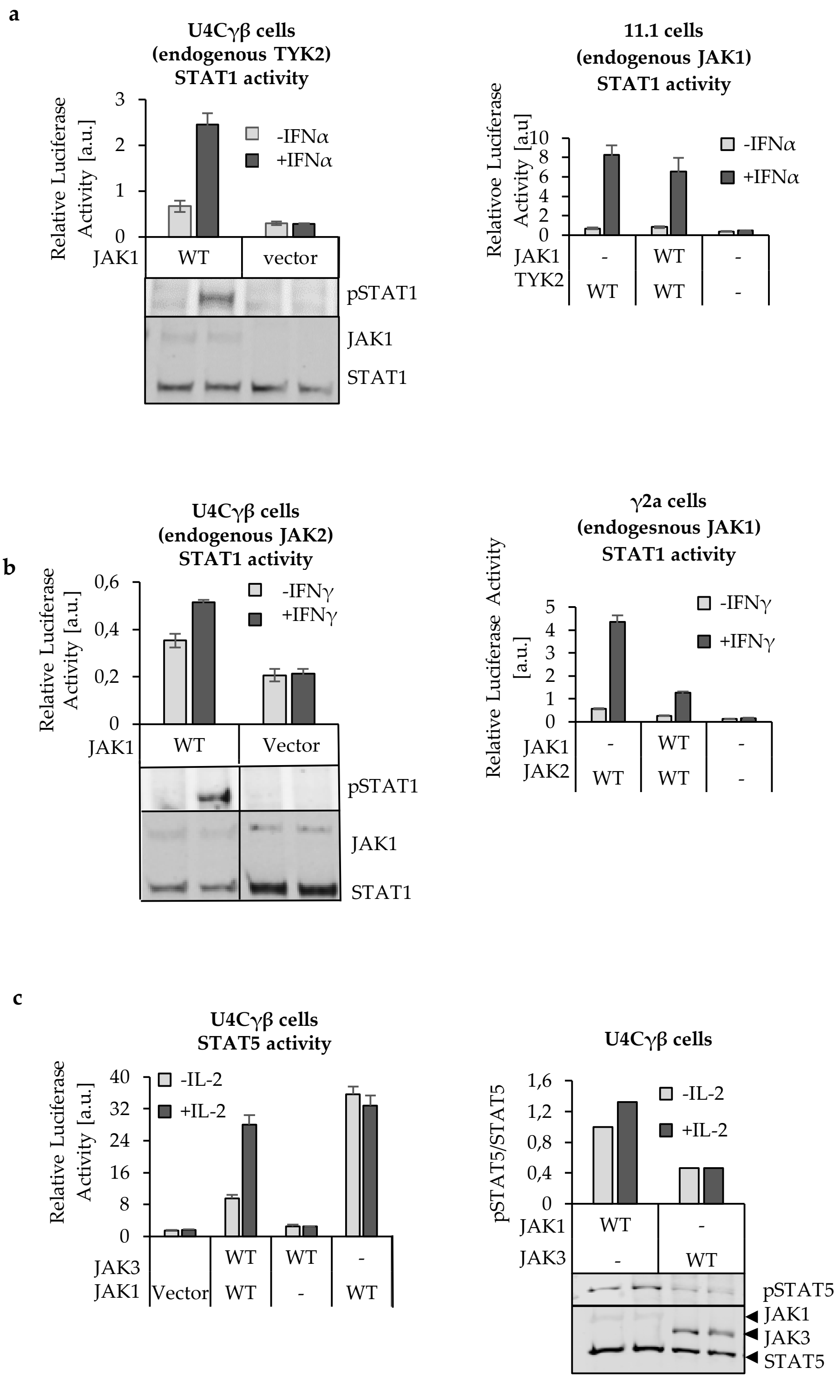

Figure S1. Both JAKs are required for cytokine responsive STAT activation in IFNα, IFNγ and IL-2 systems. (a) U4Cγβ or 11.1 cells were transiently transfected with WT JAK1 or TYK2, respectively, or with vector-only sample. IFNa responsive ISRE-Luc reporter was co-transfected to detect the STAT1 activation and pRK-TL. Neither JAK1-deficient U4Cγβ cells or TYK2-deficient 11.1 cells could respond to IFNa stimulation without transfecting the JAK that was not endogenously expressed (indicated in the headings). Errors are SD of triplicates. Cell lysates from U4Cγβ cells were immunolabeled with HA (JAK1), STAT1 and pSTAT1. The pSTAT1 status correlated with the activity data. (b) U4Cγβ cells or JAK2-deficient γ2A cells were transiently transfected with JAK1 or JAK2, respectively. IRF-GAS, IFNγ specific luciferase plasmid was used to detect STAT1 transcriptional activity and the values normalized with pRL-TK values. Errors show the SD of triplicate samples. Cell lysates from U4Cγβ cells were immunolabeled with HA (JAK1), STAT1 and pSTAT1 and the pSTAT1 status correlated with the luciferase data. (c) U4Cγβ cells were used to analyse the IL-2 systems similarly as above. Spi-Luc2 vector was used to detection of the STAT5 transcriptional activity. Right: an immunoblotted cell lysates and analysis of the pSTAT5/STAT5 ratios -/+ IL-2 with only JAK1 or JAK3 transfected. For pSTAT5 analysis, HA-tagged STAT5 was co-transfected.

Supplementary Figure 2

a

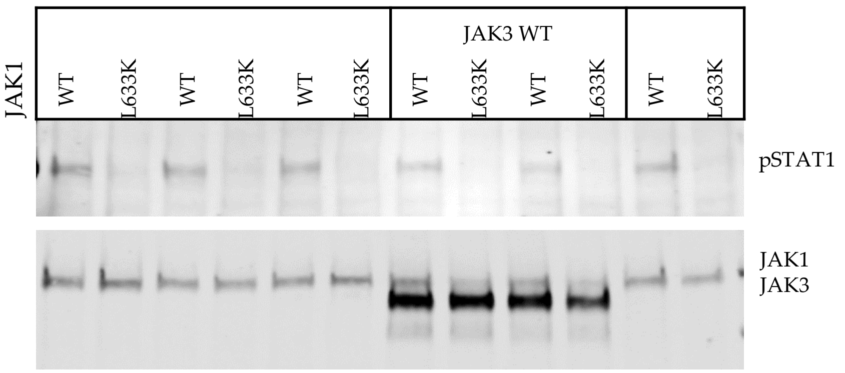

b

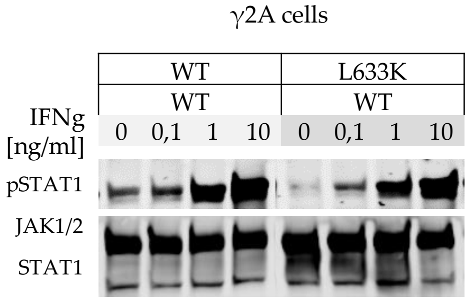

JAK1	WT				L633K			
JAK2	WT				WT			
	0	0,1	1	10	0	0,1	1	10
HA (JAK1/JAK2)	3890	3550	3090	3600	3980	4680	4750	3700
STAT1	486	497	633	658	482	616	817	310
pSTAT1	183	234	1080	2870	61,8	179	721	2090

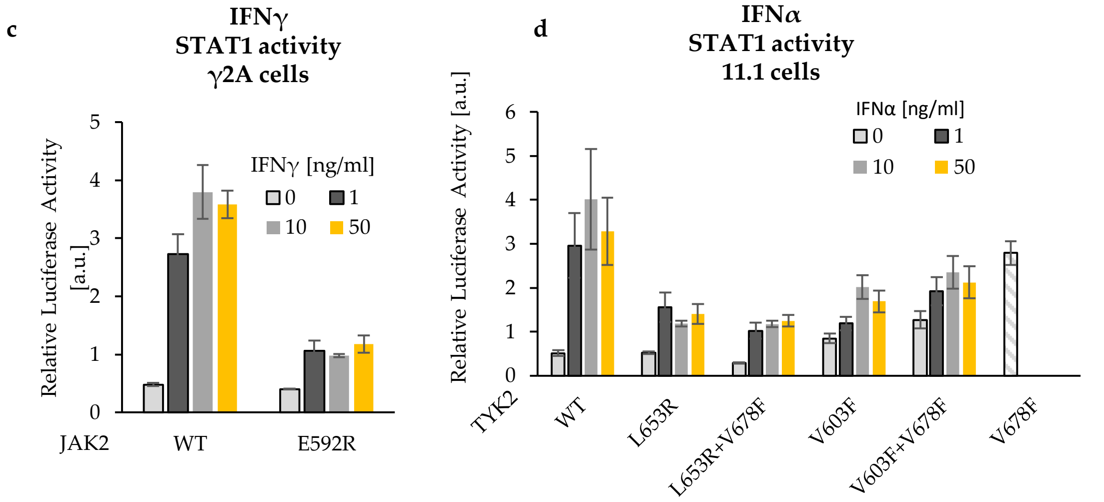

Figure S2. Modulating JH2 domain can suppress WT and hyperactive JAKs. (a) pSTAT1 analysis and expression test for Figure 2. JAK1 L633K reduces basal pSTAT1 as seen with STAT-activity assay in Figure 2B. (b) JAK1 L633K reduces basal, but not (effectively) the IFNγ stimulated pSTAT1. The effect seen with JAK1-deficient U4Cγβ cells was also apparent in JAK2- deficient γ2A cells (in JAK1-overexpression system). Cells were transfected with WT JAK2 and the L633K JAK1 and titrated with increasing amount of IFNγ. (c) JAK2 homolog strongly reduces IFNγ signaling. IRF-GAS plasmid was used to detect STAT1 activity. Average of triplicate samples (with SD) are shown. Data correlates with previous reports [Hammarén et al. 2019] (d) Homologous TYK2 JH2 αC (L653R)- and ATP-site (V603F) mutations used in this work inhibit IFNα response and constitutive activity *in cis*. ISRE-Luc plasmid was co-transfected with pTK-RL to detect relative IFNα-driven STAT1 activity. Errors are SD of triplicate samples.

Supplementary Figure 3

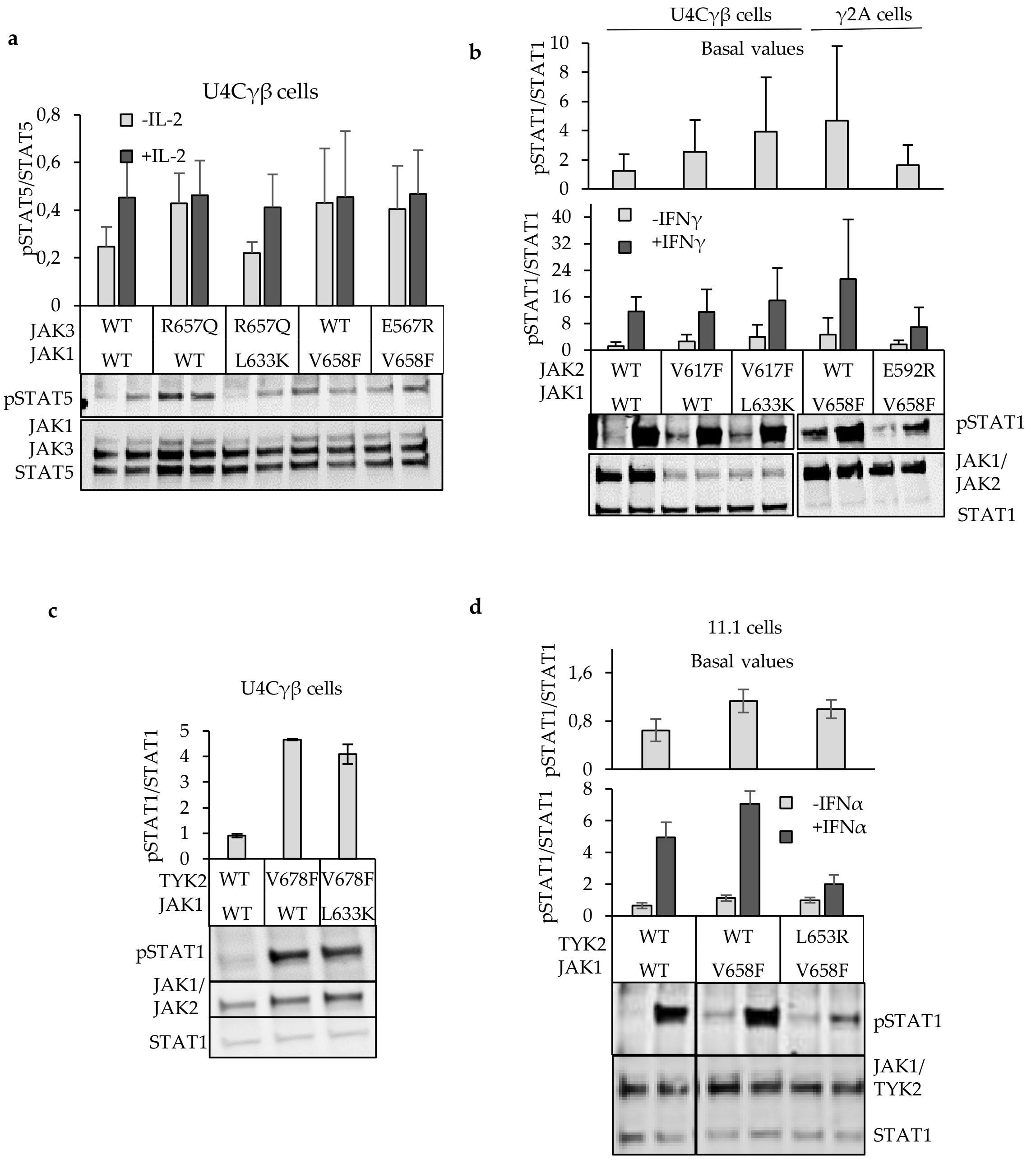

Figure S3. Effects of the αC-mutants in trans. (a) JAK1 L633K effectively inhibits JAK3-driven constitutive pSTAT5, while JAK3 homolog have no effect towards JAK1 V658F. HA-tagged JAK1, JAK3 and STAT5 were transiently transfected into U4Cγβ fibroblasts and stimulated with IL-2 or only starved. pSTAT5/STAT5 ratios were analyzed and the graph shows the averages and errors as SD of triplicate samples. (b) U4Cγβ or γ2A cells were transiently transfected with JAK1 L633K or homologous JAK2 mutant and their inhibitory potential was tested against V617F or homologous JAK1 mutant -/+ IFNg stimulation. Cell lysates were labelled with HA, STAT1 and pSTAT1 and the graph shows the pSTAT1/STAT1 average and SD from triplicate samples. The upper graph shows only the basal values from the lower graph. (c) U4Cγβ cells were transfected with TYK2 V678F and with WT JAK1 or JAK1 L633K. pSTAT1 from the un-stimulated cell lysates were analyzed, but no reduction of V678F driven hyperactivation could be detected. Errors are SD of triplicate samples. (d) TYK2- deficient 11.1 fibroblast cells were transiently transfected with JAK1 V658F and L653R TYK2, or WT JAKs, and the pSTAT1 analyzed from IFNa stimulated (or basal) cell lysates. The graph shows the average of triplicate samples with SDs. Only the basal values are shown in the upper graph.

Blots from Figure 1a

IFNγ[U4Cγβ

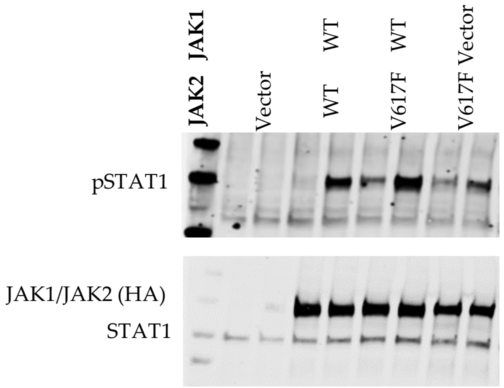

JAK2	Vector		WT		V617F		V617F	
JAK1	Vector		WT		WT		Vector	
HA (JAK1/JAK2)	10,6	71,6	2500	2220	2470	2770	2100	1690
STAT1	212	152	318	373	393	497	537	519
pSTAT1	24,2	49	74,5	762	276	1070	279	417
pSTAT1/STAT1	0,11	0,32	0,23	2,04	0,70	2,15	0,52	0,80

IFNα[U4Cγβ]

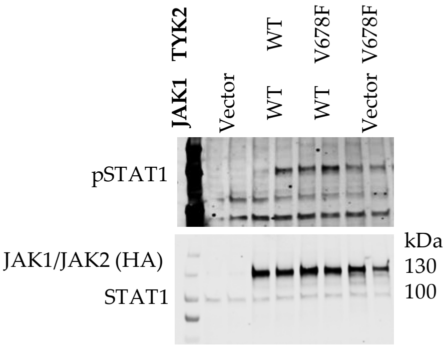

TYK2	Vector		WT		V678F		V678F	
JAK1	Vector		WT		WT		Vector	
HA (JAK1/TYK2)	12	29,7	1420	1090	1710	1080	1330	686
STAT1	136	128	135	153	200	141	155	90,1
pSTAT1	8,96	27,3	59,2	194	167	181	86,8	67,3
pSTAT1/STAT1	0,07	0,21	0,44	1,27	0,84	1,28	0,56	0,75

IFNγ [γ2A]

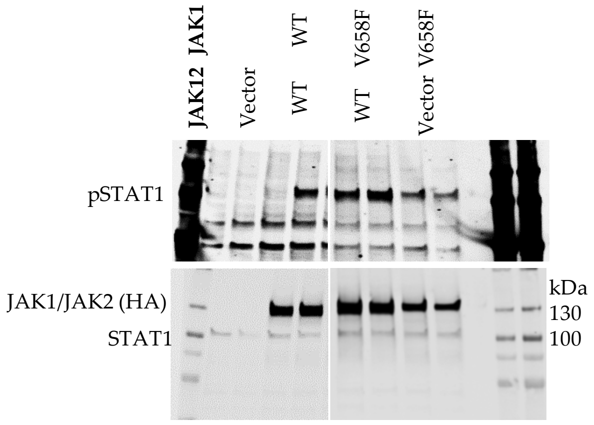

JAK1	Vector		WT		V658F		V658F	
JAK2	Vector		WT		WT		Vector	
HA (JAK1/JAK2)	0,878	3,43	1300	1380	2020	1670	1030	803
STAT1	379	6,6	33,4	196	236	339	267	114
pSTAT1	86,9	33,5	98,8	67,6	158	63,3	144	99,3
pSTAT1/STAT1	4,36	0,20	0,34	2,90	1,49	5,36	1,85	1,15

Blots from Figure 1b

IL-2 [U4Cγ]

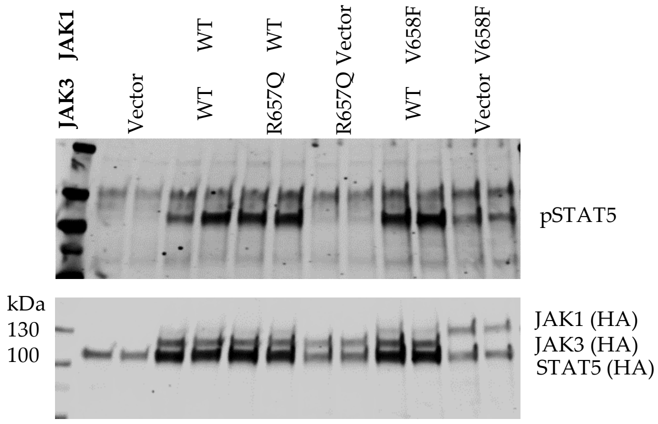

**JAK3**	Vector		**WT**		R657Q		R657Q		WT		Vector	
**JAK1**			**WT**		WT		Vector		V658F		V658F	
**JAK3**	1,13	0,208	131	88,6	128	112	114	106	164	137	-6,03	-4,57
**JAK1**	-0,841	-0,239	34,9	25,6	25,8	47,3	17,9	16,3	69,3	43,1	51,8	49,2
**STAT5**	156	118	377	372	580	412	162	168	496	504	165	142
**pSTAT5**	26,3	18	131	307	332	333	34,3	45,4	321	409	130	117
**pSTAT5/STAT5**	0,17	0,15	0,35	0,83	0,57	0,81	0,21	0,27	0,65	0,81	0,79	0,82

Blots from Figure 1c

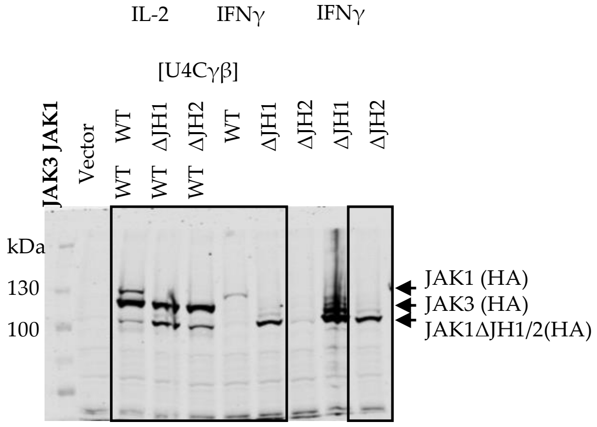

JAK1	WT	ΔJH1	ΔJH2	WT	ΔJH1	ΔJH2
**JAK3**	WT	WT	WT	-	-	-
**JAK1 ΔJH1/2**	58,8*	460	165	17,9	575	504
**JAK3 (HA)**	1190	725	867	23,8	48,8	41,2
**JAK1 WT (HA)**	178	28,3	57,2	72,7	17,2	18,8

* Unspecific band

Blots from Figure 3b

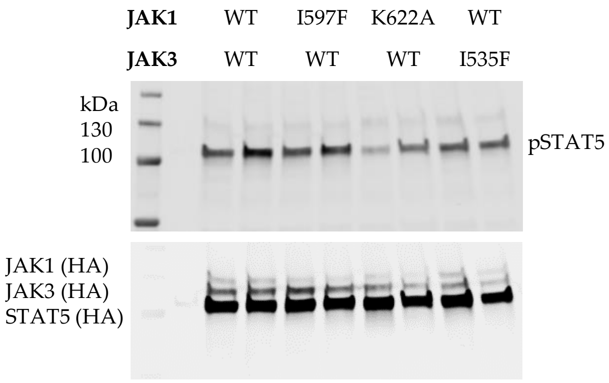

**JAK1**	WT		I597F		K622A		WT	
**JAK3**	WT		WT		WT		I535F	
**JAK1 (HA)**	753	701	535	400	367	331	763	542
**JAK3 (HA)**	1100	1200	1480	1140	859	674	745	954
**STAT1**	8530	8570	8490	8580	10200	7020	11500	6840
**pSTAT1**	10100	13300	9140	11600	3100	7640	9580	8660
**pSTAT1/ STAT1**	1,2	1,6	1,1	1,4	0,3	1,1	0,8	1,3

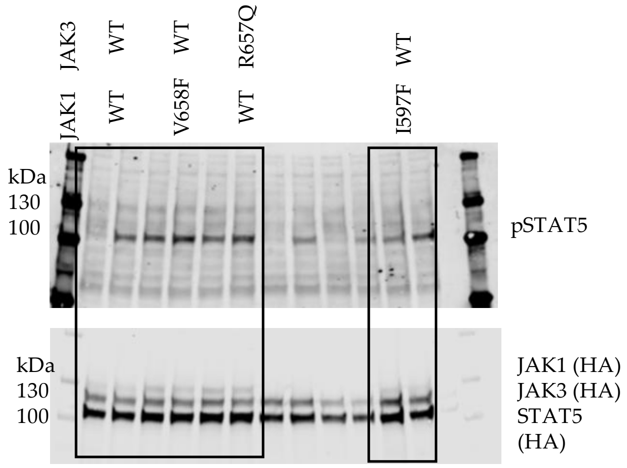

**JAK3**	WT		WT		R657Q		WT	
**JAK1**	WT		V658F		WT		I597F	
**STAT5**	1200	1240	1790	1540	1700	1790	1710	1450
**JAK3**	446	366	464	457	431	461	735	608
**JAK1**	108	92	130	123	150	127	93,9	94,3

Blots from Figure 4a

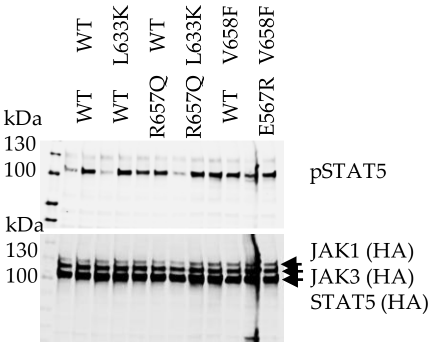

JAK1<br>JAK3	WT<br>WT		L633K<br>WT		WT<br>R657Q		L633K<br>R657Q		V658F<br>WT		V658F<br>E567R	
**JAK1**	255	404	361	449	387	295	255	503	576	543	2500	795
**JAK3**	1030	1340	1130	1530	1250	1100	830	1330	1090	868	4080	1200
**STAT5**	6610	9010	10900	13200	7810	7730	7420	10900	9320	7240	7480	8350
**pSTAT5**	1890	6810	1410	8530	4610	5860	1670	7920	7290	6870	5640	7750
**pSTAT5/STAT5**	0,29	0,76	0,13	0,65	0,59	0,76	0,23	0,73	0,78	0,95	0,75	0,93

Blots from Figure 4b

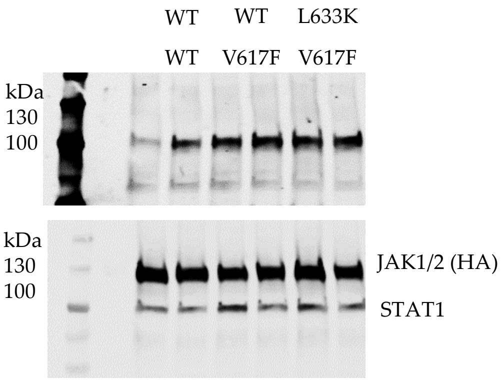

JAK2<br>JAK1	WT<br>WT		V617F<br>WT		V617F<br>L633K	
**JAK1/JAK2 (HA)**	2800	2390	2120	2120	3100	1980
**STAT1**	539	440	650	718	801	745
**pSTAT1**	176	406	571	725	686	907
**pSTAT1/STAT1**	0,33	0,92	0,88	1,01	0,86	1,22

Blots from Figure 4c

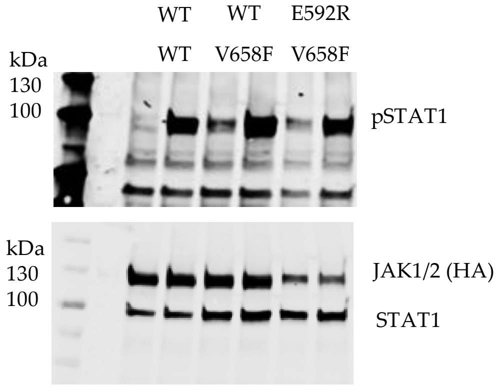

JAK1<br>JAK2	WT<br>WT		V658F<br>WT		V658F<br>E592R	
**JAK1/JAK2 (HA)**	1810	1780	1650	1660	575	611
**STAT1**	714	772	1370	1620	1010	1190
**pSTAT1**	0,16	2,41	0,24	1,91	0,19	0,83

Blots from Figure 5a

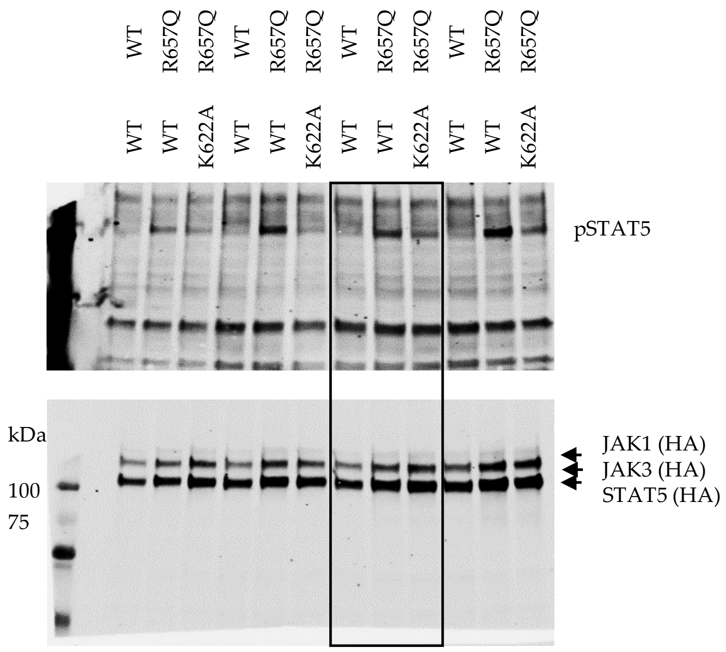

**JAK1**	**WT**	**R657Q**	**R657Q**	**WT**	**R657Q**	**R657Q**	**WT**	**R657Q**	**R657Q**	**WT**	**R657Q**	**R657Q**
**JAK3**	**WT**	**WT**	**K622A**	**WT**	**WT**	**K622A**	**WT**	**WT**	**K622A**	**WT**	**WT**	**K622A**
**STAT5**	541	514	263	386	285	183	276	349	218	301	199	145
**pSTAT 5**	90,5	188	62,6	87,9	144	79,2	72,9	167	73,1	63,3	89,3	24,7
**JAK1**	9,17	12,9	16,7	12,3	26,4	18,5	15,1	25,4	21,8	17,8	35,9	54,4
**JAK3**	53	79,7	116	71,7	126	97,6	68,6	111	129	87,4	170	170

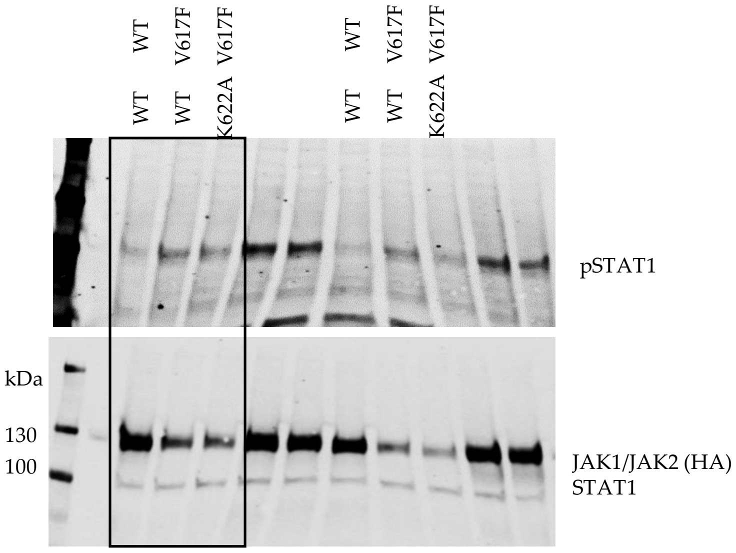

**JAK2**<br>**JAK1**	**WT**<br>**WT**	**V617F**<br>**WT**	**V617F**<br>**K622A**	**WT**<br>**WT**	**V617F**<br>**WT**	**V617F**<br>**K622A**
**HA (JAK1/JAK2)**	484	100	62,6	319	75,9	40
**STAT1**	18,2	13,1	18,8	13,4	12,7	8,21
**pSTAT1**	33,3	48,3	38,3	25,2	64	15,4
**pSTAT1/STAT1**	1,8	3,7	2,0	1,9	5,0	1,9

Blots from Figure 5b

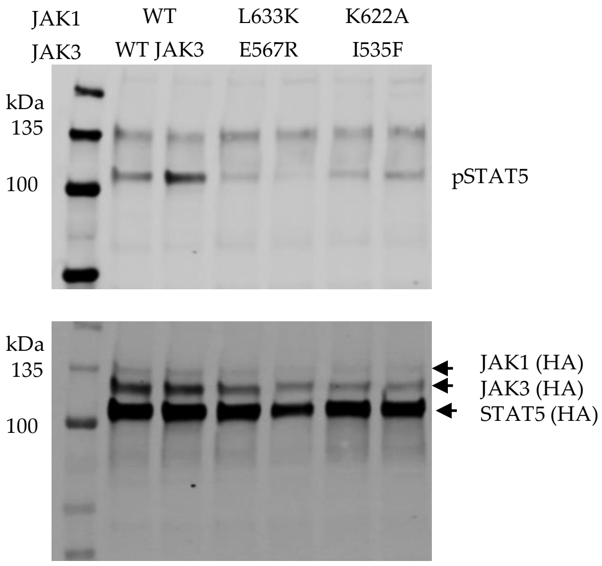

**JAK1**	**WT**		**L633K**		**K622A**	
**JAK3**	**WT JAK3**		**E567R**		**I535F**	
**JAK1**	59,9	44,8	26,2	19,3	21,4	21
**JAK3**	206	196	125	68,2	75,7	68,8
**STAT5**	1440	1550	1190	468	1350	1120
**pSTAT5**	697	1160	247	120	327	405
**pSTAT5/STAT5**	0,5	0,7	0,2	0,3	0,2	0,4
